# Menstrual, fertility and psychological impacts after uterine compression sutures for postpartum hemorrhage: a prospective cohort study

**DOI:** 10.1186/s12884-023-05530-8

**Published:** 2023-03-29

**Authors:** Lee Ting Kwong, Sai Fun Wong, Po Lam So

**Affiliations:** Department of Obstetrics and Gynecology, Tuen Mun Hospital, Tuen Mun, Hong Kong

**Keywords:** Uterine compression sutures, B-lynch suture, Hayman suture, Cho suture, Pregnancy, Menstruation, Fertility, Psychological impact

## Abstract

**Background:**

Uterine compression suture is an important conservative surgical technique in managing atonic postpartum hemorrhage. In this study, we aim to evaluate the subsequent menstrual, fertility and psychological outcomes after uterine compression sutures.

**Methods:**

This was a prospective cohort study between 2009 and 2022 conducted in a tertiary obstetric unit (6000 deliveries per year) in Hong Kong SAR. Women with primary postpartum hemorrhage successfully treated with uterine compression sutures were followed-up in postnatal clinic for two years after delivery. Data on menstrual pattern were collected during each visit. Psychological impact after uterine compression suture was assessed using a standardized questionnaire. Subsequent pregnancies were identified by territory-wide computer registry and telephone interviews. Women with postpartum hemorrhage treated with uterotonic agents only were chosen as controls.

**Results:**

In our cohort (n = 80), 87.9% of women had return of menses within six months after delivery. Regular monthly cycle was observed in 95.6% of women. Majority of women reported similar menstrual flow (75%), menstrual days (85.3%) and no change in dysmenorrhea status (88.2%) as compared before. Among eight (11.8%) women who reported hypomenorrhea after uterine compression sutures, two cases of Asherman’s syndrome were diagnosed. Among 23 subsequent pregnancies (16 livebirths), no significant differences in outcome were observed except more omental or bowel adhesions (37.5% vs. 8.8%, *p* = 0.007), recurrence of hemorrhage (68.8% vs. 7.5%, *p* < 0.001) and repeated compression sutures (12.5% vs. 0%, *p* = 0.024) were seen in women with previous compression sutures. Over half of the couple declined future fertility after uterine compression sutures with 38.2% of women recalled unpleasant memories and 22.1% reported life-long adverse impact especially tokophobia.

**Conclusion:**

Majority of women with history of uterine compression sutures had similar menstruation and pregnancy outcomes as compared to those who did not have sutures. However, they had higher intrapartum risk of visceral adhesions, recurrence of hemorrhage and repeated compression sutures next pregnancy. Furthermore, couple could be more susceptible to negative emotional impact.

**Supplementary Information:**

The online version contains supplementary material available at 10.1186/s12884-023-05530-8.

## Background

Uterine atony is the commonest etiology of primary postpartum hemorrhage (PPH), ranging from 30 to 80% worldwide [1]. In the event of failed hemostasis with uterotonic agents, early recourse to conservative surgical management is advisable to prevent maternal morbidity and mortality [2]. Since 1997, various techniques of uterine compression sutures were described to treat atony, such as B-lynch suture, Hayman suture and Cho suture [3–5]. Being relatively easy and quick to perform, these sutures were efficacious in preventing 97% of hemostatic hysterectomies [6]. In our previous publication, 75% of hysterectomies were prevented in women treated with uterine compression sutures with additional second-line hemostatic procedures [7]. Overall, the short-term complication rate was low.

While uterine compression sutures were considered effective and safe, literatures on long-term menstruation and fertility outcomes were scarce. The fertility rate after different sutures ranged from 11 to 75% [6]. Limiting by small number of women included, these studies suggested most pregnancies were uncomplicated and carried till term [8–11]. Nevertheless, rare complications of fundal uterine rupture at third trimester and placenta accreta spectrum overlying the rupture site were reported which raised the concern of whether localized uterine necrosis after compression sutures might increase the risk of myometrial weakening and abnormal placental implantation [12–16].

Menstrual outcome is another important indicator of fertility preservation, especially in women with no desire for future pregnancy. Limited available studies suggested no significant change in menstrual pattern was observed after compression sutures with 91.5 to 100% of these women reported return of menstruation within eight months after delivery [17, 18]. Nevertheless, these data might subject to recall bias as most studies were retrospective.

Psychological impact among women with PPH without hysterectomy is often neglected. Women described the experience as ‘a feeling of powerlessness’ and ‘a fear of bleeding until death takes over’ [19, 20]. Sentilhes reported two-third of women with pervasive negative memory and fear of death after PPH [19]. In addition, 5.9% of women reported sexual problems and 60% suffered from intense anxiety during their next pregnancy [19]. Up till now, there has been no research investigating the psychological impacts of both women and their partners after uterine compression sutures for PPH.

In the present study, we aim to determine any change in menstrual pattern, adverse pregnancy outcomes and psychological impact in women after uterine compression sutures.

## Methods

This was a prospective cohort study conducted in Tuen Mun Hospital, Hong Kong SAR over a 13-year period. The inclusion criteria were women delivered with primary PPH (blood loss > = 500ml) successfully treated with uterine compression sutures between January 2009 to June 2022 who consented to this study. We performed uterine compression suture as the first line surgical treatment for woman undergoing caesarean section with uterine atony after failing standard uterotonic agents (oxytocin, ergometrine, carbetocin, carboprost and misoprostol). The types of compression sutures performed would be at obstetrician’s discretion. In case of unsuccessful hemostasis, uterine artery ligation was performed for further devascularization, followed by intraoperative uterine artery embolization by radiologist if woman was hemodynamically stable, or hysterectomy if she was unstable. In case of hemorrhage from placenta previa, we performed uterine artery ligation as the first line surgical treatment for devascularization, followed by compression suture if there remained localized placental site bleeding. As for atonic PPH after completion of caesarean section or vaginal delivery, more conservative management including intrauterine balloon tamponade and uterine artery embolization performed in interventional radiology suite were considered. Principles outlined in the Declaration of Helsinki were followed and this study was approved by the New Territories West Cluster Research Ethics Committee, Hospital Authority, Hong Kong SAR.

All compression sutures were applied by specialists who had regular training on obstetric emergencies. B-lynch suture, Hayman suture and Cho suture were included in our study design and surgical techniques were strictly followed [3–5]. Monocryl-1 (polyglecaprone 25) absorbable suture was used for B-Lynch and Hayman suture while Vicryl-1 (polyglactin 910) was used for Cho suture. Uterine artery ligations were performed with Vicryl-1 (polyglactin 910) suture. Bakri balloon (Cook Medical, US) was used as intrauterine balloon tamponade. Uterine artery embolization was performed by radiologist intraoperatively in theatre or in interventional radiology suite, involving injection of absorbable gelatin sponges to extravasating uterine vessels for devascularization. Women were excluded from the present study if hemostatic hysterectomies were performed.

Baseline characteristics and clinical details of women were identified through both clinical notes and electronic patient record system. The baseline psychological status of women was assessed by the Edinburgh Postnatal Depression Scale (EPDS), which is a questionnaire conducted by adding together the scores of 10 items, indicating the severity of depressive symptoms.

### Menstrual and psychological assessment

Women were assessed in postnatal clinic at six weeks, four months, one year and two years after delivery. Informed consent was obtained. During each visit, they were enquired about the mode of feeding, contraception, cessation of lochia, return of menstruation and a full menstrual history. Primary endpoints were defined as regular cycle, no change in menstrual flow, no change in menstrual days, no dysmenorrhea or no change in the severity of dysmenorrhea as compared to before. In case of menstrual abnormalities, gynecological examination, pregnancy test, cervical smear, ultrasound of pelvis (GE Healthcare Voluson) and endometrial biopsy with or without diagnostic hysteroscopy (KARL STORZ) were performed to rule out structural causes. Genital swabs and serum hormone level (estradiol, follicle-stimulating hormone, prolactin and thyroid hormone) were performed if clinically indicated. In the last session of the follow-up, women were assessed on their psychological impact using a standardized questionnaire designed by Sentilhes [19].

### Subsequent pregnancy outcomes

In June 2022, we identified all subsequent pregnancies of these women delivered in any public hospitals in Hong Kong SAR through territory-wide electronic registry system. To identify any subsequent pregnancies delivered in private sector or outside our territory, all women received telephone interviews and a list of questions relating to their pregnancies outcomes were asked. A standardized script was available for obtaining consent over telephone. Control group was identified by the next five consecutive women who suffered from atonic PPH, successfully treated with uterotonic agents and had subsequent pregnancies. Women in both groups were matched by their order of pregnancies, number and mode of deliveries. Women with any other previous surgeries were excluded. The demographics and clinical details of these women were retrieved in the same manner as study group.

Baseline characteristics of subsequent pregnancies including age of women, interpregnancy interval and order of pregnancy were collected. Number of subsequent ectopic pregnancies, miscarriages, terminations, pregnancies beyond 24 weeks gestation, hypertensive disease, placenta previa, placenta accreta, preeclampsia and uterine rupture were compared. Gestation at delivery, birth weight, numbers of small-for-gestational age fetuses, operative findings, recurrence of atonic PPH and number of repeated compression sutures were compared between the two groups.

### Statistical analysis

SPSS Statistics version 21 (IBM, Armonk, NY) was used. Categorical and continuous variables were presented as n (%) and median (range) respectively. Pearson chi-square test and Fisher’s exact test were used for comparing dichotomous data while Student’s *t*-test and Mann-Whitney *U* test were used for comparing continuous data. A two-sided *P* value less than 0.05 was considered statistically significant.

## Results

### Study participants

Over the 13-year study period, 90 uterine compression sutures (0.1%) were performed in the background of 80,087 deliveries. After excluding 10 women who had hysterectomies performed due to failed hemostasis, 80 women were eligible for this study (Figure S1). The baseline characteristics of women with uterine compression sutures were shown in Table 1. Most participants were primiparous young women with singleton pregnancy. All women delivered by cesarean sections with all compression sutures performed at the same operation. 86.3% of uterine compression sutures were applied for uterine atony. There were nine postpartum short-term complications identified, including four hematometra requiring drainage, four endometritis treated with antibiotics and one retained product of conception requiring surgical evacuation. Majority (97.5%) of women had no antecedent psychiatric disorders with a median Edinburgh Postnatal Depression Scale (EPDS) score of three at their sixth week appointment. Seventy-six (95%) of them had EPDS score fewer than 10.

**Table 1:** Clinical characteristics of women successfully treated with uterine compression sutures

	Uterine compression suture (n = 80)
Age (years)	33 (24–53)
Singleton pregnancy	66 (82.5)
Multiparity	27 (33.8)
Hypertensive disease	5 (6.3)
Diabetes mellitus	12 (15)
Psychological disorder	2 (2.5)
Placenta previa	10 (12.5)
Placenta abruptio	6 (7.5)
Gestation (weeks)	38 (25–41)
Indication for suture	
Uterine atony	69 (86.3)
Placenta previa	11 (13.8)
Detail of procedures	
B-lynch	19 (23.8)
Hayman	27 (33.8)
B-lynch + Hayman	1 (1.3)
B-lynch + UAL	10 (12.5)
Hayman + UAL	19 (23.8)
Cho + UAL	2 (2.5)
B-lynch + UAL + IUBT	1 (1.3)
Hayman + UAL + UAE	1 (1.3)
Blood loss (mL)	2800 (500-10000)
Birth weight (g)	3060 (795–4470)
Postpartum complications	
Hematometra	4 (5)
Pyometra	0
RPOC	1 (1.3)
Endometritis	4 (5)
Uterine necrosis	0
EPDS	3 (0–17)

*UAL*, uterine artery ligation; *Cho*, Cho suture; *IUBT*, intrauterine balloon tamponade; *UAE*, uterine artery embolization; *RPOC*, retained product of conception; *EPDS*, Edinburgh Postnatal Depression Scale

Data are expressed as n (%) or median (range)

### Menstrual outcome and psychological impact

Sixty-eight (85%) out of 80 women were compliant to our prospective assessment. The median follow-up period was 12 months (range 6–20 months). Of those who were not on exclusive breastfeeding, 87.9% of women reported return of menses within six months after delivery at a median of nine weeks (Table 2). Of note, all participants had return of menses by the end of their last visits. Regular monthly cycle was observed in 95.6% of women with over three quarters of them reporting similar menstrual flow (75%), menstrual days (85.3%) and no change in dysmenorrhea status (88.2%) when compared to before.

**Table 2:** Menstrual outcomes of women after uterine compression sutures

	Women with prospective follow-up (n = 68)
Exclusive breastfeeding	10 (14.7)
Resumption of menses < 6 months Ϯ	51 (87.9)
Return of menses after delivery (weeks) Ϯ	9 (4–36)
Regular monthly cycle	65 (95.6)
Menstrual flow	
No change in flow	51 (75)
Hypomenorrhea	8 (11.8)
Workup completed*	8 (100)
Asherman’s syndrome	2 (25)
Mild synechiae	1 (12.5)
Perimenopause	1 (12.5)
Menorrhagia	9 (13.2)
Workup completed*	5 (55.6)
Menstrual days	
No change in days	58 (85.3)
Shortened	3 (4.4)
Workup completed*	2 (66.7)
Perimenopause	1 (50)
Lengthened	7 (10.3)
Workup completed*	6 (85.7)
Dysmenorrhea	
No/ No change	60 (88.2)
Increased	5 (7.4)
Workup completed*	5 (100)
Decreased	3 (4.4)
Workup completed*	3 (100)

ϮWomen on exclusive breastfeeding were not included

*Ultrasound of pelvis, endometrial investigation +/- blood test for hormone level

Data are expressed as n (%) or median (range)

Eight (11.8%) women reported hypomenorrhea after uterine compression sutures. Among them, two (25%) were diagnosed with Asherman’s syndrome (Figure S2), one (12.5%) with mild synechiae and one (12.5%) delivered at age 53 was perimenopausal. None of them had future fertility wish. Among other women experiencing changes in menstrual pattern, workup did not reveal any structural causes.

Regarding assessment on psychological impact, all women completed questionnaires at the end of their last visits (Table 3). Among them, 72.1% declined any more fertility wish with 26.5% did so due to fear of recurrence of PPH. Importantly, 38.2% of women recalled unpleasant memories especially fear of pain and death; and 22.1% of them reported life-long adverse impact especially tokophobia (73.3%). All participants denied any long-term effects on sexual function or marital relationship. When women were asked about their partners’ views on the delivery episodes, 54.4% of them recalled unpleasant memories and 51.5% declined any wish for future pregnancy.

**Table 3:** Psychological impact on couple after uterine compression sutures

	Frequency (n = 68)
1. I plan to get pregnant again.	
Yes	19 (27.9)
No	49 (72.1)
Cause(s)	
Already completed family	17 (34.7)
Personal health issue	15 (30.6)
Fear of recurrence of PPH	13 (26.5)
2. Do you have any unpleasant memory of the experience of PPH?	
Yes	26 (38.2)
Cause(s)	
Fear of death	10 (38.5)
Fear of pain	12 (46.2)
Fear of separation from baby	4 (15.4)
No	42 (61.8)
3. Are there any long-term adverse impacts in your life after PPH?	
Yes	15 (22.1)
Cause(s)	
Tokophobia	11 (73.3)
Phobia of hospital	3 (20)
Phobia when seeing blood	3 (20)
Fear of death	3 (20)
Sexual dysfunction	0
Marital problem	0
No	53 (77.9)
4. Does your partner have any unpleasant memory after the index pregnancy?	
Yes	37 (54.4)
No	31 (45.6)
5. Does your partner wish for further child after the index pregnancy?	
Yes	33 (48.5)
No	35 (51.5)

*PPH*, postpartum hemorrhage. Data are expressed as n (%)

### Subsequent pregnancy outcomes

All women responded to our telephone interviews. None of them reported infertility. Among the 19 (27.9%) women who expressed wishes of getting pregnant again in postnatal clinic, 17 (89.5%) of them conceived. Among all 23 subsequent pregnancies, there were 16 livebirths, four miscarriages, two terminations due to fetal anomaly and one ectopic pregnancy (Table 4). All pregnancies were conceived naturally. Of the 16 singleton livebirths, there were no cases of preeclampsia, placenta accreta spectrum or uterine rupture. The median gestational age, term delivery, preterm delivery, stillbirth, birth weight and the proportion of small-for-gestational-age fetuses were comparable between both study and control groups. All women with previous uterine compression sutures decided to have repeated cesarean sections in their next pregnancies. Concerning operative finding, there were significant higher risk of omental or bowel adhesions to the uterus in women with previous uterine compression sutures (37.5% vs. 8.8%, *p* = 0.007). Eleven out of 16 women suffered from recurrence of atonic PPH (68.8% vs. 7.5%, *p* < 0.001) with a median blood loss of 800mL compared with 250mL in the control group (*p* < 0.001). Two women in the study group failed medical treatment requiring repeated uterine compression sutures for hemostasis (12.5% vs. 0%, *p* = 0.024). No peripartum hysterectomies were performed in our cohort.

**Table 4:** Subsequent pregnancy outcomes of women with or without uterine compression sutures

	Uterine compression suture (n = 23)	Control (n = 90)	*P* value
Age (years)	32 (26–39)	33 (21–44)	0.937
Interpregnancy interval (months)	36 (5-108)	36 (10–120)	0.483
Ectopic pregnancy	1 (4.3)	0 (0)	0.204
Miscarriage	4 (17.4)	7 (7.8)	0.260
Termination of pregnancy	2 (8.7)	3 (3.3)	0.268
Term pregnancy	14 (60.9)	69 (76.7)	0.126
Preterm delivery	2 (8.7)	11 (12.2)	0.636
Stillbirth	0 (0)	0 (0)	> 0.999
Hypertensive disease	0 (0)	4 (5)	> 0.999
Diabetes mellitus	2 (12.5)	16 (20)	0.728
Placenta previa	1 (6.3)	0 (0)	0.167
Fibroid	1 (6.3)	6 (7.5)	0.861
Polyhydramnios	1 (6.3)	2 (2.5)	0.619
Pre-delivery hemoglobin (g/dL)	11.3 (8.3–14.4)	10.8 (8.5–13.4)	0.139
Pre-delivery platelet (10^9^/L)	202 (116–315)	180 (130–300)	0.185
Duration of labor (hour)	0 (0)	0 (0)	> 0.999
Gestation (weeks)	38 (36–40)	38 (29–41)	0.257
Birth weight (kg)	2.85 (2.30–3.59)	3.05 (1.30–3.80)	0.212
Small-for-gestational age fetus	1 (6.3)	2 (2.5)	0.425
Caesarean section	16 (100)	80 (100)	
Operative finding			
• Groove over uterus	1 (6.3)	0 (0)	0.167
• Omental/ bowel adhesion	6 (37.5)	7 (8.8)	0.007
Blood loss (mL)	800 (200–1400)	250 (100–1000)	< 0.001
• Recurrence due to uterine atony	11 (68.8)	6 (7.5)	< 0.001
Repeated uterine compression suture	2 (12.5)	0 (0)	0.024

*PPH*, postpartum hemorrhage

Values are expressed as n (%) or median (range)

## Discussion

### Main findings

Our study demonstrated majority of women had return of menstruation with similar patterns after uterine compression sutures. Two cases of Asherman’s syndrome and one mild uterine synechiae were diagnosed among eight women with hypomenorrhea. None of our subjects contemplating next pregnancy reported infertility. Among the 23 subsequent pregnancies, despite most outcome parameters were comparable between women in both study and control groups, we observed a higher incidence of omental or bowel adhesion, recurrence of atonic PPH and repeated compression sutures in the study group. A significant proportion of women were reluctant for future pregnancy after compression sutures with more than one-fifth of them admitting unpleasant memories and long-term adverse impact.

### Strengths and limitations

The main strength in our study is the two-year longitudinal clinical follow-up with high patients’ compliance. Thus, clinical information was recorded prospectively minimizing recall bias. In addition, by retrieving pregnancy outcomes from both electronic system and telephone interviews, we improved our data quality in terms of accuracy and exhaustiveness by including early pregnancy loss and terminations which might not had been managed in public sectors. Apart from pregnancies, we also investigated other indicators of subsequent fertility including couple’s fertility wish, workup of any subfertility and return of menstruation. Most of the previous publications included women with B-lynch sutures while there have been reports suggesting probable higher rate of complications after Cho sutures or when compression suture was combined with pelvic vessel ligation [21]. Thus, another distinct advantage is that we included a variety of compression sutures and a combination with other uterine sparing techniques. Our study is also the first which reported the psychological impact of women after uterine compression sutures.

One of our limitations is all of our women with compression sutures had it performed during caesarean section and none at relaparotomy or after vaginal delivery. Furthermore, we did not record the decision time of compression sutures from the start of operation which could have affected the subsequent outcomes. Another pitfalls in our study is the lack of comparison of menstrual and psychological outcomes with those in control group. One of our limitations is the potential underestimation of women with mild asymptomatic uterine synechiae as the indication of hysteroscopy was symptom-driven. In addition, since the last assessment ranged from six months to 20 months postpartum, the results of women’s psychological assessment could be influenced by a change in psychological status with time. Moreover, we did not take into account of women’s social support, socioeconomic status and newborn’s condition which could have affected the psychological outcomes of them. Finally, since we did not invite the partners for direct interview, their responses were indirectly collected from our participants.

### Interpretation

Uterine compression sutures are easier, quicker and cost-effective to perform comparing with other uterine conservation techniques. Overall, data on long-term pregnancy outcomes has been reassuring [2, 8–11]. Nevertheless, most of the literatures were case reports or small case series.

Poujade O et al. reported uterine synechiae in one-fourth of their cohort which is higher than our result [22]. It is likely that our study might have underestimated women with mild asymptomatic uterine synechiae as hysteroscopy was offered only in symptomatic women. In our study, there were two women with Asherman’s syndrome and one mild uterine synechiae. Both of our women with Asherman’s syndrome had Hayman sutures applied during their elective cesarean sections. Their immediate post-operative course was unremarkable. The first woman with uterine atony secondary to amniotic fluid embolism also had uterine artery ligations and later pelvic vessel embolization six hours after operation due to persistent bleeding. The procedure was performed by radiologist with gelatin sponge particles in interventional radiologist suite. We postulated the severe hemodynamic shock and the combination of three devascularization techniques might have contributed to uterine ischemia and scarring over the endometrium. The second woman had caesarean sections performed due to cephalopelvic disproportion. Hayman sutures and uterine artery ligations were performed due to uterine atony. Another woman with mild uterine synechiae had elective caesarean sections performed complicating with uterine atony requiring Hayman’s sutures. Presenting with pelvic pain at her second week after delivery, hematometra was diagnosed requiring drainage twice. We agree with other authors that compression sutures might potentiate the formation of endometrial scarring and ischemic damage by excessive suture tension on the myometrium [23]. Moreover, hematometra and uterine synechiae might be more prevalent after Hayman and Cho sutures as they directly obliterate the anterior and posterior uterine walls. In order to prevent endometrial scarring, application of appropriate tension on uterine walls by suture material which is firm, monofilament and quickly absorbed is of paramount importance. Recently, there were reports of novel compression sutures which were removed in early postoperative period [24]. Nevertheless, the sample size was small and further evaluation on long-term outcome is needed before clinical application.

Our subsequent pregnancy rate was 29% which corresponds well with previous studies [6, 11]. Pregnancies from three months to ten years after uterine compression sutures have been reported [25]. Similarly, in the present study, the interpregnancy interval was between five months to nine years. Comparing with control group with no compression sutures performed, we did not identify any differences in the rate of preterm birth and small-for-gestational-age fetus. Regarding mode of delivery, literatures reported successful vaginal deliveries in up to one-third of the cases [8]. Interestingly, none of our women chose vaginal delivery. Taking into account of their response in the psychological interviews, their negative memories and fear of recurrence of PPH might explain their wish of having the deliveries conducted in a relatively more controlled manner. We observed significantly more atonic PPH (68.8%) and repeated compression sutures (12.5%) in our cohort. Similar findings were reported by Fuglsang who observed 15% of subsequent deliveries required repeated B-Lynch sutures or hysterectomies [8]. In addition, significantly more omental or bowel adhesions were noted in one-third of our cohort. Hence, our results suggest a higher level of anticipation of PPH and surgical difficulty should be considered in subsequent deliveries. Previous studies reported severe complications of uterine rupture in next pregnancies [11–13]. In our cohort, we reported none but one woman with evidence of grooves over the uterus which was likely an ischemic defect suggestive of excessive pressure on the myometrium. The number of women with uterine grooves is likely to be underreported as these features could only be diagnosed during operation. Therefore, high vigilance of uterine dehiscence or rupture should be maintained in any women presenting with abdominal pain antenatally. Overall, with the small sample size, it is difficult to draw a conclusion about the effect of compression sutures on subsequent pregnancy outcomes.

Remarkably, 72.1% of women and 51.5% of their partners were reluctant for future pregnancy. Nearly one-fourth of them and over half of their partners reported negative memories afterwards. Significantly, among those with long-term adverse impact after the delivery episodes, 73.3% had tokophobia. With our result, we suggest involving professional input in debriefing and providing psychological support sensitively to couple would be beneficial in early postnatal period. Conducting a longer period of research is required to assess the impact to their long-term quality of life.

## Conclusions

Majority of women with history of uterine compression sutures had similar menstruation and pregnancy outcomes as compared to those who did not have sutures. In their future pregnancies, despite most pregnancies were carried till term uneventfully, a higher risk of women with atonic PPH, repeated compression sutures, omental and bowel adhesions were observed. Professional input may be beneficial to address couple’s traumatic memory and emotional recovery after delivery episodes. Large-scale study is needed to narrow this risk estimate.

## Electronic supplementary material

Below is the link to the electronic supplementary material.

**Additional file 1: Figure S1.** Study population

**Additional file 2: Figure S2.** Asherman’s syndrome

## Data Availability

The datasets used and/or analyzed during the current study are available from the corresponding author on reasonable request.

## Abbreviations

PPH: Postpartum hemorrhage
EPDS: Edinburgh Postnatal Depression Scale
