# Supplementary material for: Menstrual, fertility and psychological impacts after uterine compression sutures for postpartum hemorrhage: a prospective cohort study
Source: BMC Pregnancy Childbirth. 2023 Mar 29;23:217. doi: 10.1186/s12884-023-05530-8 (PMC10053948; doi:10.1186/s12884-023-05530-8)
Supplement: Supplementary file 1 — Additional file 1: Figure S1. Study population [file 12884_2023_5530_MOESM1_ESM.docx]

Figure S1. Study population

Total deliveries 80,087

Uterine compression sutures, n=90

Excluded peripartum hysterectomy, n=10

Women included in the study, n=80

Women lost to follow-up, n=12

Prospective follow-up for menses pattern and psychological impact, n=68

Women with fertility wish, n=19

Subsequent pregnancy, n=23
