# Supplementary figures and images for: Menstrual, fertility and psychological impacts after uterine compression sutures for postpartum hemorrhage: a prospective cohort study
Source: BMC Pregnancy Childbirth. 2023 Mar 29;23:217. doi: 10.1186/s12884-023-05530-8 (PMC10053948; doi:10.1186/s12884-023-05530-8)

Figure S2. Asherman’s syndrome


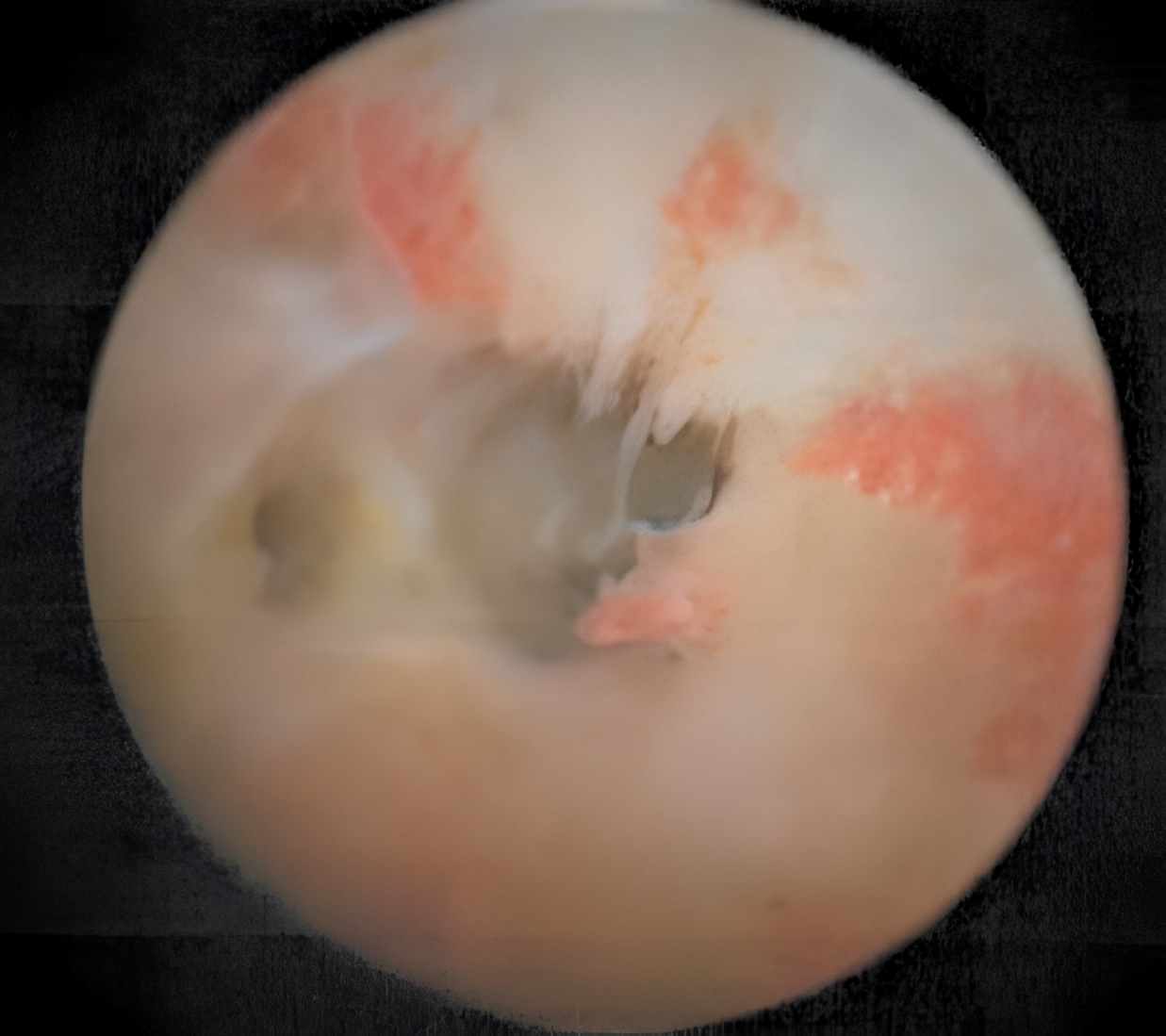

Supplement: Supplementary file 2 — Additional file 2: Figure S2. Asherman’s syndrome [file 12884_2023_5530_MOESM2_ESM.docx]
